# Differences in and verification of genetic alterations in chemotherapy and immunotherapy for metastatic melanoma

**DOI:** 10.18632/aging.203640

**Published:** 2021-10-21

**Authors:** Yang Li, Yuling Gao, Weiling Chu, Jianjian Lv, Zhi Li, Tongxin Shi

**Affiliations:** 1 Department of Dermatology, The Affiliated Qingdao Municipal Hospital of Qingdao University, Qingdao, Shandong, China; 2 Department of Oncology, The Affiliated Qingdao Municipal Hospital of Qingdao University, Qingdao, Shandong, China

**Keywords:** metastatic melanoma, prognostic factor, chemotherapy, immunotherapy, resistance-related genes, genetic alterations

## Abstract

Background: Metastatic melanoma has poor therapeutic response and may present resistance to chemotherapy or immunotherapy. Significant differences are observed in the survival time of patients with metastatic melanoma based on the administration of chemotherapy or immunotherapy; thus, we have explored the important role of specific differential genes between the two therapies in their effect on treatment response in melanoma.

Methods: Metastatic melanoma gene expression data (RNAseq, mutation and methylation) and patient clinical information were downloaded from The Cancer Genome Atlas database and grouped according to chemotherapy or immunotherapy. The differentially expressed genes of the two groups were further screened for signature genes through a protein–protein interaction network and Lasso-Cox regression model. Then, differences in the treatment response, overall survival, mutation and methylation of characteristic genes were compared. Finally, western blot and real-time qPCR technology were used to detect the expression differences of the signature genes in metastatic melanoma tumor tissues in patients undergoing chemotherapy and immunotherapy.

Results: The overall survival of the chemotherapy-based treatment group was significantly higher than that of the immunotherapy-based group. The immune infiltration level of immature dendritic cells (DCs) in the chemotherapy group was significantly higher than that in the immunotherapy group. Finally, seven signature genes were selected: *CCKBR, KCNJ11, NMU, MMP13, ITGA10, IGFBP1* and *CEACAM5*. The results of these signature genes were significantly differentiated between the chemotherapy and immunotherapy groups in terms of overall survival and disease progression in response to treatment. In addition, differences in the expression of these genes were verified by western blot and real-time qPCR.

Conclusion: In this study, significant differences in the expression of signature genes were verified. The findings indicate that immature DCs with potential application value should be considered and high mutation sites of signature genes should be identified to reduce the occurrence of treatment resistance.

## INTRODUCTION

Although malignant metastatic melanoma accounts for less than 5% of skin tumors, it causes the highest number of skin cancer-related deaths [1]. Thus, choosing the best treatment options is important. In recent years, an in-depth understanding of the pathogenesis of metastatic melanoma and the importance of the anti-tumor immune response and regulatory mechanism have provided melanoma patients with new treatment opportunities, which include targeted drug therapy and immune checkpoint inhibitors [2]. Nonetheless, chemotherapy remains the primary treatment for metastatic melanoma, and the inherent resistance of melanoma cells and incomplete response to chemotherapy drugs are major problems associated with melanoma treatment [3].

Many studies have explained the mechanism of gene mutation and methylation modification in the process of melanoma chemotherapy resistance [4, 5]. However, there are many artificial interference factors in the design of these studies, which cannot replicate the actual changes in tumors that occur in the natural environment and the final outcome of patients. There is also a lack of relevant reports on metastatic melanoma chemoresistance-related genes. Studies have shown that while chemotherapy kills tumor cells, it also stimulates the surrounding normal cells to release a chemical substance that can stimulate tumor cell growth and eventually lead to treatment resistance [6–8]. Therefore, exploring differences in gene expression in metastatic melanoma between chemotherapy and immunotherapy is not only helpful for improving our understanding of chemotherapy or immunotherapy resistance genes but also has guiding significance for the research and development of targeted therapy drugs.

## MATERIALS AND METHODS

### Gene expression data acquisition and screening

Data were collected according to the rules of public data use on official websites, and all normalized data were collected from The Cancer Genome Atlas data center (TCGA, https://portal.gdc.cancer.gov). Patients were screened based on the administration of chemotherapy (e.g., dacarbazine, cisplatin, etc.,) and immunotherapy (e.g., IL-2, interferon, etc.,). The exclusion criteria included samples with *BRAF, NRAS, NF1*, and *KIT* gene mutations and patients who were lost to follow-up.

### Differential gene analysis and screening

After completing the grouping of samples (chemotherapy and immunotherapy), the DESeq2 package (version 1.24) of R software (version 4.02, R Foundation for Statistical Computing, Vienna, Austria) was used to analyze the differences in gene expression between the two groups. The filter conditions for significant differences in gene expression were |log2FC| >1.5 and *P* < 0.05. Then, the STRING protein–protein interaction database was used to screen the key differential genes [9], and the filtering conditions in the R package STRINGdb (version 2.30) were set to score >500 and connectivity >2.

Finally, the key screening method, Lasso regression and univariate Cox prognostic model were used. Due to the collinear relationship between the expression of certain genes, the constructed prognostic model exhibited overfitting. Lasso’s penalty coefficient (λ) was used to filter out the collinearity factors, and only the most representative factors were retained for the establishment of the prognosis model. The degree of Lasso regression complexity adjustment was controlled by the parameter λ. A greater value of λ corresponds to a greater penalty to obtain a model with fewer variables from a complex model. In this study, the parameter λ value was set to 1, and the cutoff value was set to 0.6 in the R package glmnet (version 4.02).

### Analysis of the significance of signature genes

This study focuses on the biological significance of the final screening of key genes and the difference in responsiveness to chemotherapy and immunotherapy. In addition, the mutation status and methylation changes of the signature genes were also influencing factors of chemotherapy tolerance. According to the clinical information, the chemotherapy and immunotherapy patients were matched based on the barcodes of patient samples in TCGA database. Therefore, this study also analyzed data on the mutation status, methylation changes and site information of signature genes, and all the data were downloaded from the TCGA database. R packages maftools [10] (version 2.0) and Gviz [11] (version 1.28) were used to compare gene mutation differences and map methylation site information, respectively.

### Verification of signature genes

According to the standards of the Ethics Review Committee of Qingdao Municipal Hospital and the conditions of informed consent provided by the patients, skin tissue samples from twenty patients with metastatic MM who underwent chemotherapy and immunotherapy and ten normal patients were collected. According instructions of the western blotting techniques [12], relative protein concentration of signature genes in tissues was detected. All antibody reagents were purchased from Abcam (Abcam.com). The SYBR Green labeling method was used to detect the gene expression level of signature genes by real-time qPCR [13], and the gene amplification primer sequence was obtained from PrimerBank [14]. ABI StepOne Plus was used to calculate the relative expression differences of signature genes. The primers used in this study are listed in Supplementary Table 1.

### Quantification and statistical analysis

The western blot results of the signature genes were quantitatively analyzed by Quantity One (version 4.6.2, Bio-Rad Laboratories, Inc.,). The function of this tool can convert the gray value of the western blot image into data for comparative analysis. The real-time qPCR results were converted using the 2^−ΔΔct^ method to calculate the amplification fold changes, and Student’s *t*-test was used to test whether the differences between the two groups were statistically significant (*P* < 0.05).

## RESULTS

### Chemotherapy achieved a better overall survival expectancy

**Figure 1:**
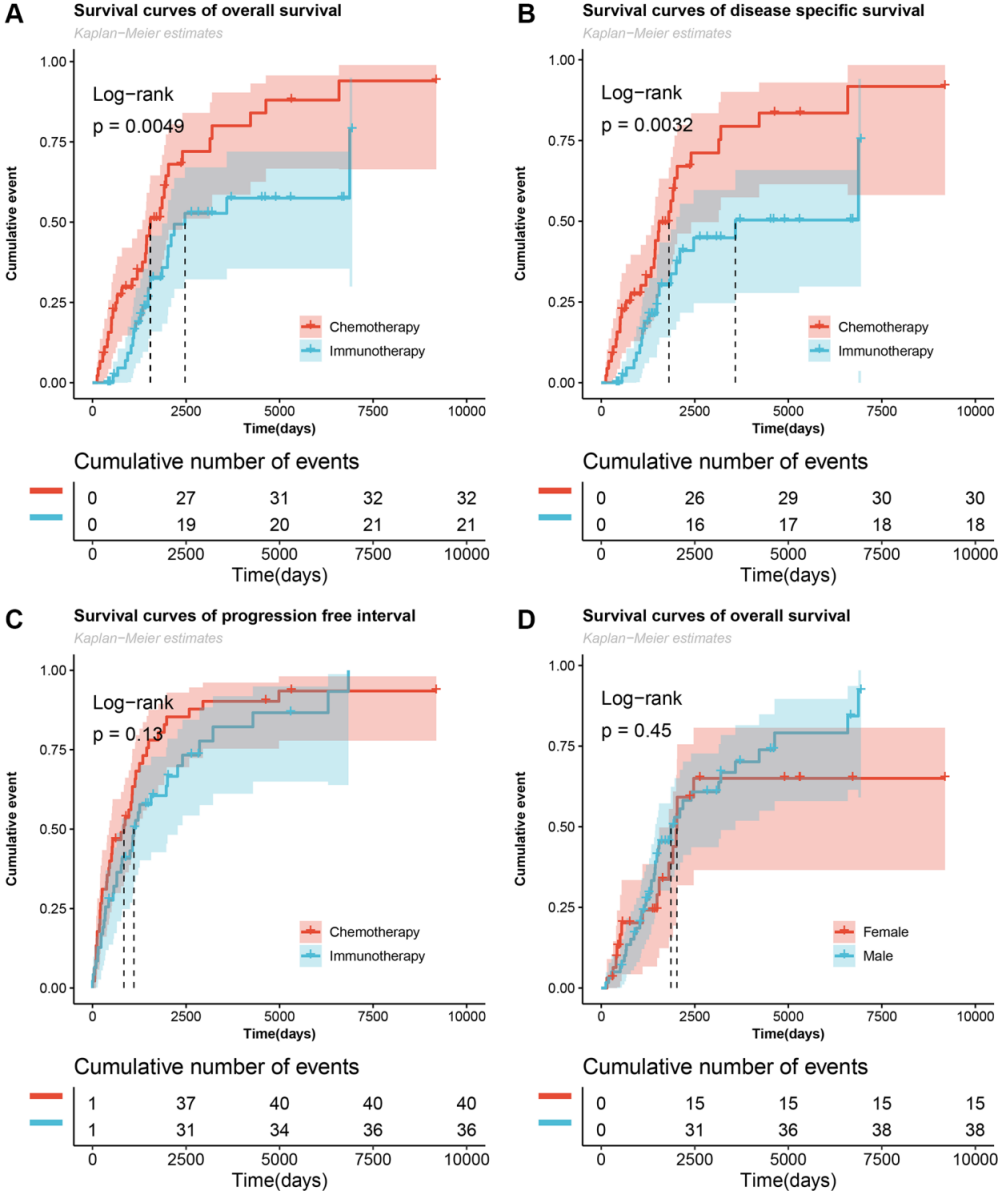
**Cumulative number of survived events in patients with metastatic melanoma undergoing chemotherapy and immunotherapy.** (**A**–**C**) The Log-rank test for overall survival, disease specific survival, and progression-free interval survival curves of the chemotherapy and immunotherapy groups. (**D**) Survival curves of overall survival of male and female patients.

According to the screening rules, the gene expression data of 46 patients with metastatic melanoma treated with chemotherapy and 47 patients with immunotherapy were ultimately used in this study. Table 1 shows the differences in the treatment period and response to chemotherapy or immunotherapy. The results showed that there was a significant difference in the response rate between the chemotherapy and immunotherapy patients. In addition, taking the final survival status (death or survival) as the outcome indicator, the cumulative overall survival and disease-specific survival events of patients in the chemotherapy group were significantly higher than those in the immunotherapy group (Figure 1A and 1B), whereas significant differences were not observed in progression-free survival (Figure 1C). The results also showed that there was no difference in overall survival between male and female patients (Figure 1D).

**Table 1:** Characteristics of chemotherapy and immunotherapy patients.

**Group**	**Chemotherapy**	**Immunotherapy**	***P* (Stat)**	**Effect Size (CI)^#^**
Observations (*N*)				
	46	47		
Gender % (*N*)				
FEMALE	33% (15)	36% (17)	0.89@ (0.02)	0.86 (0.33; 2.2)
MALE	67% (31)	64% (30)		
missing	0% (0)	0% (0)		
Age at diagnosis (year)				
Mean (SD)	55 (16)	51 (12)	0.65^$^ (0.16)	0.28 (–0.14; 0.7)
valid (missing)	45 (1)	45 (2)		
Treatment response % (event)				
Progressive Disease	46% (21)	21% (10)	**0.035@** **(8.6)**	0.39 (0.15; 0.6)
Complete Response	17% (8)	15% (7)		
Partial Response	4.3% (2)	6.4% (3)		
Stable Disease	0% (0)	11% (5)		
missing	33% (15)	47% (22)		
Days drug therapy (days)				
Mean (SD)	75 (86)	238 (233)	**<0.001^$^** **(0.46)**	–0.94 (–1.4; –0.45)
valid (missing)	38 (8)	35 (12)		
OS % (event)				
Yes	28% (13)	55% (26)	**0.019@** **(5.5)**	0.33 (0.13; 0.84)
No	70% (32)	45% (21)		
missing	2.2% (1)	0% (0)		
OS.time (days)				
Mean (SD)	1.8e + 03 (1.8e + 03)	2.4e + 03 (1.9e + 03)	0.24^$^ (0.22)	–0.34 (–0.76; 0.085)
valid (missing)	45 (1)	44 (3)		
DSS % (event)				
Yes	33% (15)	62% (29)	**0.012@** **(6.3)**	0.31 (0.12; 0.79)
No	65% (30)	38% (18)		
missing	2.2% (1)	0% (0)		
DSS.time (days)				
Mean (SD)	1.8e + 03 (1.8e + 03)	2.4e + 03 (1.9e + 03)	0.24^$^ (0.22)	–0.34 (–0.76; 0.085)
valid (missing)	45 (1)	44 (3)		
PFI % (event)				
Yes	11% (5)	23% (11)	0.2@ (1.6)	0.41 (0.1; 1.4)
No	87% (40)	77% (36)		
missing	2.2% (1)	0% (0)		
PFI.time (days)				
Mean (SD)	1.3e + 03 (1.7e + 03)	1.5e + 03 (1.6e + 03)	**0.33^$^** **(0.2)**	–0.14 (–0.56; 0.28)
valid (missing)	45 (1)	45 (2)		

^#^Effect size is calculated by Cohen’s d statistics. ^@^*P* value and statistic result are calculated by Chi-sq.test. ^$^*P* value and statistic result are calculated by ANOVA. Abbreviations: CI: confidential interval; OS: overall survival; DSS: disease specific survival; PFI: progression free interval.

### Anti-tumor properties of signature genes

**Figure 2:**
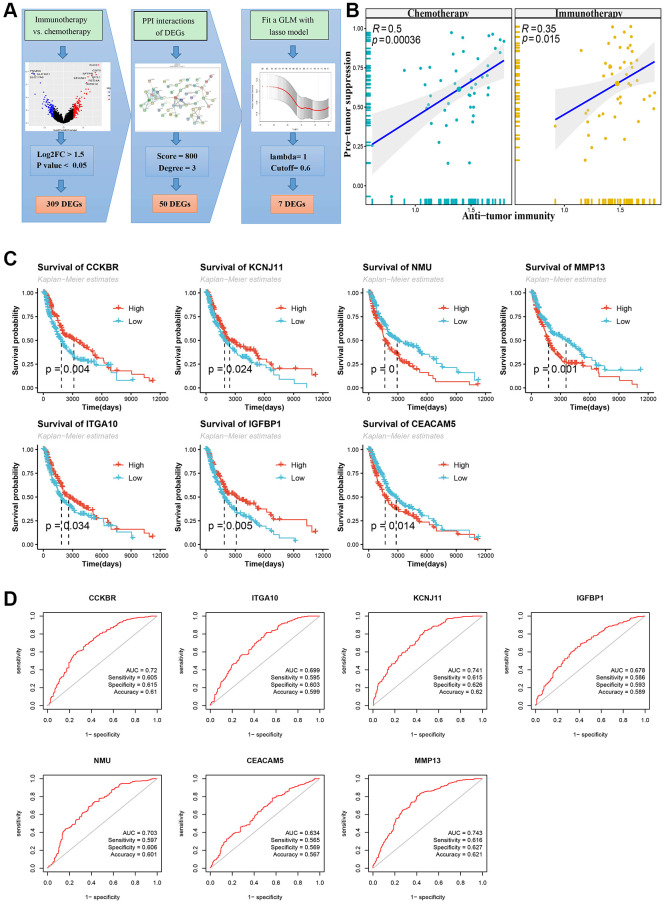
**Flowchart of screening signature genes and its Kaplan-Meier survival curve and receiver operating characteristic curve (ROC).** (**A**) Flowchart of screening signature genes. Including volcano plot of differentially expressed genes (DEGs), DEGs’ PPI network and Lasso-COX fitting model. (**B**) DEGs immune cell infiltration analysis, Pearson correlation test of Anti-tumor and Pro-tumor suppression based on immune cell type enrichment scores. (**C**) Log-rank test for survival curves of signature genes. (**D**) ROC curve, area under curve (AUC), sensitivity, specificity and accuracy values of signature genes.

Figure 2A shows the screening flowchart of signature genes, and based on |log2FC| > 1.5 and *P* < 0.05, 309 DEGs were used to construct the PPI protein interaction network. Subsequently, the genes in the marginal region were filtered out according to score >500 and degree >2 of the protein molecule. In this result, 50 genes (Supplementary Table 2) were used to construct a generalized linear Lasso-Cox regression model to further remove genes with collinear characteristics. Finally, seven signature genes were obtained: *CCKBR, KCNJ11, NMU, MMP13, ITGA10, IGFBP1* and *CEACAM5*.

According to The Cancer Immunome Atlas (TCIA) [15], a tumor immune cell infiltration analysis was performed, and the enrichment scores of these DEGs showed significant tumor suppressive effects in both the chemotherapy group and the immunotherapy group (Figure 2B). A comparison of the immune cell infiltration scores between the two groups showed that the immature dendritic cells (DCs) were significantly higher in the chemotherapy group than the immunotherapy group (*P* < 0.01), while the central memory CD8 T cells and type 2 T helper cells were significantly higher in the immunotherapy group than the chemotherapy group (*P* < 0.05) (Supplementary Figure 1A). The Pearson correlation test between immunoinfiltrating cells is shown in the attached Supplementary Figure 1B. In addition, these seven signature genes were divided into high and low groups according to their median expression levels. Significant anti-tumor effects were observed based on these groupings, and the high expression group of *CCKBR* and *KCNJ11* showed better anti-tumor effects (Supplementary Figure 1C).

### Functional enrichment in the PPI network

The functional enrichment analyses of the 50 genes screened by the PPI network included gene ontology terms (cell composition, molecular function and biological process), pathways (KEGG and Reactome) and protein domains (InterPro and Ffam database). (Supplementary Figure 2A). The biological functions and effects of these key genes are shown in Supplementary Figure 2. The cell composition category mainly includes cell membrane synapses, keratin filaments, collagen trimers, cell junctions and ion channel complexes (Supplementary Figure 2B). The molecular function category mainly includes ion channel activity and receptor binding, such as cytokine activity and receptor binding function, chemokine activity and receptor binding function, collagen and hormone binding function, etc., (Supplementary Figure 2C). The enrichment results of the integrated protein domains were mainly conserved sites of CXC chemokines, chemokine IL-8-like superfamily, hemopexin superfamily and intermediate filament (Supplementary Figure 2E). The signaling pathways mainly included IL-17, TNF and estrogen signaling, ECM, cytokine-cytokine and neuroactive-ligand receptor interactions, and nicotine and morphine addiction (Supplementary Figure 2F).

### Differences in the survival of signature genes

As shown in Figure 2C, patients with high expression of the signature genes *CCKBR, KCNJ11, ITGA10*, and *IGFBP1* had higher overall survival prognoses while patients with low expression of *NMU, MMP13*, and *CEACAM5* had higher overall survival. This finding implies that patients with longer overall survival periods are positively correlated with anti-tumor scores for certain signature genes with high expression (*CCKBR, KCNJ11, ITGA10*, and *IGFBP1*) and other signature genes with low expression (*NMU, MMP13* and *CEACAM5*). However, based on the anti-tumor properties described above, this property was significant only in the signature genes *CCKBR* and *KCNJ11* (Supplementary Figure 1).

Then, receiver operating characteristic curves (ROCs) of these signature genes were drawn, and the area under the curve (AUC) was used to indicate the predictive value of the model. As shown in Figure 2D, the AUC values of the CCKBR, KCNJ11, NMU and MMP13 genes were greater than 0.7 and the AUC values of the other signature genes were between 0.7 and 0.6. In addition, the sensitivity, specificity and accuracy of the seven characteristic genes were also calculated to evaluate the predictive value of the signature genes in the ROC model.

### Differences in mutations, methylation and treatment response

**Figure 3:**
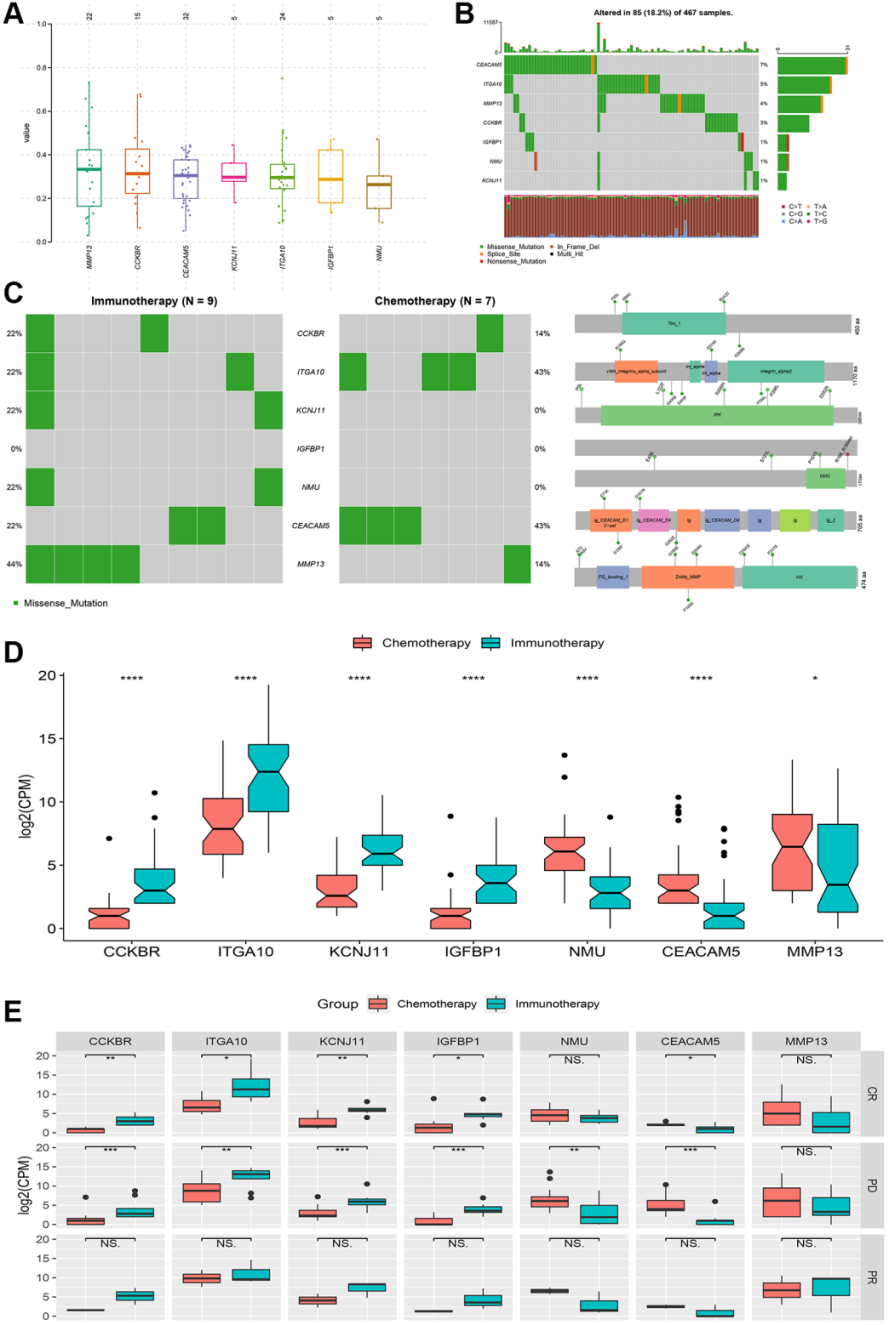
**Genetic variation and relative expression differences of signature genes between chemotherapy and immunotherapy.** (**A** and **B**) Mutation frequency and type of signature genes. (**C**) Mutations and protein domain differences of signature genes between chemotherapy group and immunotherapy group. (**D**) Differences in relative expression levels (log2(CPM)) of signature genes between the two groups. (**E**) Comparison of complete response (CR), partial response (PR) and progressive disease (PD) differences in signature genes between the two groups. ^*^P < 0.05, ^**^P < 0.01, ^***^P < 0.001, ^****^P < 0.0001, NS: no significance.

The signature genes *CEACAM5, ITGA10* and *MMP13* had the highest proportion of mutations among all 467 patients with cutaneous melanoma of the skin (Figure 3A and 3B). Although the frequency of mutations in the immunotherapy group was higher than that in the chemotherapy group, no significant difference was found between the two groups because the total sample of the study was only 93 cases (Figure 3C). A comparison of the protein domains of the missense mutations showed that the protein domains of the immunotherapy group mainly occurred in the box-labeled region while those of the chemotherapy group mainly occurred outside the box-labeled region.

Treatment responses include complete response, partial response, and disease progression, which are important indicators for judging the effectiveness of tumor treatment. Based on significant differences in signature genes between the two treatment groups (Figure 3D), they were grouped according to the treatment response, and significant differences in disease progression were observed for all genes except for the *MMP13* gene and differences in complete response were mainly observed for the *CCKBR, KCNJ11, ITGA10, IGFBP1* and *CEACAM5* genes (Figure 3E). No significant differences were found for partial responses.

In addition, a group comparison was performed for methylation site changes of these signature genes. Significant differences were not found between the chemotherapy group and the immunotherapy group or between the treatment response groups. The comparative results are shown in Supplementary Figure 3.

### Expression changes of signature genes in metastatic melanoma tissues

**Figure 4:**
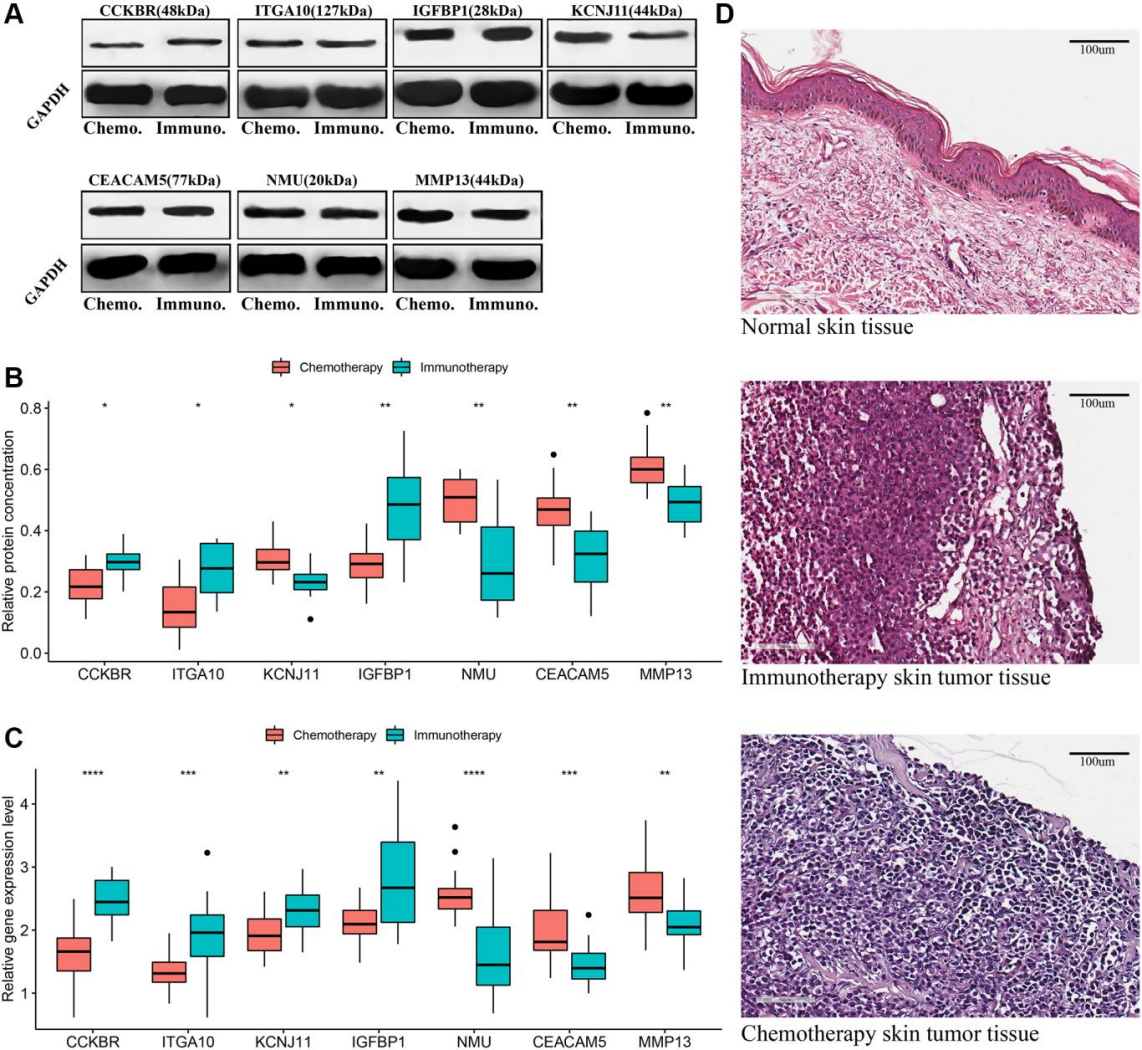
**Validation of signature genes in patients with metastatic cutaneous melanoma undergoing chemotherapy and immunotherapy.** (**A** and **B**) Western blot results of signature genes and *t*-test comparison results of gray values between the two groups. (**C**) *T*-test comparison results of the relative expression levels of signature genes in real-time qPCR between the two groups. (**D**) Hematoxylin-eosin (HE) staining: normal skin tissue, tumor tissues of metastatic melanoma patients receiving chemotherapy and immunotherapy. ^*^P < 0.05, ^**^P < 0.01, ^***^P < 0.001, ^****^P < 0.0001.

Using basic public data, this study verified the expression changes of signature genes in patients with metastatic melanoma after chemotherapy and immunotherapy. The gene expression level and protein concentration were expressed using real-time qPCR and western blotting, respectively, with reference to the relative expression level of the internal control. As shown in Figure 4A–4B, the comparison of the relative protein concentration of characteristic gene expression showed that the protein concentrations of CCKBR, ITGA10 and IGFBP1 in melanoma tumor tissue in the postoperative chemotherapy group were significantly higher than those in the immunotherapy group while the protein concentrations of KCNJ11, NMU, MMP13 and CEACAM5 in the chemotherapy group were significantly lower than those in the immunotherapy group. In addition, the relative differences in gene expression levels (Figure 4C) were consistent with the results of previous data analysis (Figure 3D). It is worth noting that the difference in protein concentration expressed by the KCNJ11 gene is opposite to the difference in gene expression level. Additionally, the hematoxylin-eosin (HE) staining results showed that the skin tissue structure of tumor patients was disordered and deeply stained compared with that of normal individuals (Figure 4D).

## DISCUSSION

It is generally believed that melanoma is an immunogenic cancer and immunotherapy, with its potential therapeutic effects, may theoretically resolve the deficiency of tumor chemotherapy resistance. However, in this study, patients with metastatic melanoma who received immunotherapy did not obtain a longer overall survival and progression-free interval and in fact experienced a higher mortality rate caused by specific diseases. In addition, studies have evaluated the use of immunotherapy based on interferon alfa-2b [16, 17] and high-dose IL-2 [18] to treat metastatic melanoma; however, the results were not as effective as in theory. Four prospective trials in Atkins’ observation study [16] showed that in patients with high-risk resection of melanoma, adjuvant therapy with high-dose interferon-alpha-2b can effectively improve the relapse-free survival rate but not the overall survival rate. Moreover, this therapeutic effect only occurred in patients older than 46 years of age in two prospective studies, and significant differences were not observed in the combined result. In the case of a combination of many melanoma treatment methods (such as cocktail therapy), in which one or a combination plays a key role, it is difficult to obtain a fixed answer under different circumstances. For example, in a randomized phase III trial of combination chemoimmunotherapy (dacarbazine, cisplatin, and interferon-alpha-2b) with or without IL-2 as adjuvant therapy, the results did not show a significant improvement in progression-free survival or the treatment response rate in patients with metastatic melanoma [19].

Similar to the differences in immune cell infiltration observed earlier in this study, immature DCs have strong antigen-phagocytosis ability and differentiate into mature DCs when they ingest antigens or are stimulated by certain factors [20], while mature DCs express high levels of costimulators and adhesion factors [21]. A recent study showed that treatment with immature dendritic cell-targeted vaccines significantly improved the survival rate in murine melanoma models, and this improved efficacy was positively correlated with tumor lysate gene expression levels and tumor-infiltrating lymphocytes (TILs) [22]. Appropriate chemotherapy is not only the main method of killing immature cells and proliferating active cells but can also produce abundant antigens to activate a large number of immature DCs. Immature DCs, as the most functional APC found thus far, can acquire the initiation ability of specific cytotoxic T lymphocytes (CTLs) under independent or dependent induction of T helper cells [23]. In addition, a DC-based immunotherapy vaccine (Sipuleucel-T) for prostate cancer was approved for use by the FDA in 2010 [24, 25].

Growing evidence has shown that tumors progress slowly over a long period of time before they manifest as a distinct disease. According to a recent study by Harvard Medical School and others, the initial cancer-causing mutation may have appeared as early as 40 years ago, at least in some cases [26]. This finding seems slightly alarmist, although it provides sufficient warning of the importance of tumor diseases. After all, many deaths are caused by tumors every year, with 1.76 million new cancer cases and 600,000 cancer deaths in the United States in 2019 [27] and an estimated 4.3 million new cancer cases and 2.9 million cancer deaths in China in 2018 [28]. Moreover, certain deaths caused by tumor diseases (such as lung cancer and colorectal cancer) have a tendency to increase annually, especially in low-income countries [29]. Detection of mutant genes and CpG methylation is of great significance to the selection of targeted therapy for patients, and it is also the main reason for immunotherapy resistance [4, 5]. Although significant differences in signature gene mutations and CpG methylation were not observed between the two treatment groups, it is undeniable that mutations and methylation occurred and may play an important role in the development of melanoma and the exploration of new treatment methods.

Tumor genesis is characterized by a series of complex and persistent environmental factors. According to the functional enrichment results of the 50 key genes screened by PPI in this study, systemic treatment not only induces changes in the basic functions and activities of cells (e.g., membrane synapses, ion channel complexes, etc.,) but also leads to abnormalities in the immune and endocrine systems (e.g., cytokines/chemokines, TNF and estrogen signaling). Subsequently, researchers have proposed a new tumor treatment method: combination therapy targeting the tumor microenvironment [30]. TIL infusion, as a new immunomodulatory strategy, was approved for clinical use by the FDA in 2019 and can effectively extend the lives of patients with advanced cancer. The number of infused cells was related to a good response. This strategy has been applied for patients with metastatic melanoma [31, 32] and can be used as an alternative therapy after the failure of immune checkpoint inhibitor therapy [33]. Solid tumors that are highly infiltrated with immune cells and proinflammatory cytokines have been classified as inflammatory tumors [34].

The tumor microenvironment is an environment composed of malignant cells, normal cells, immune components, blood vessels, ECM, and other molecules. These components can work alone and jointly to affect sensitivity to chemotherapy or immunotherapy. As new immunomodulatory treatments and technological innovations continue to advance the field of cancer immunotherapy, the goal of personalized medicine seems to have become a reality. From single reagents to systemic treatment (including physical, chemical, biological and immunotherapy), almost every treatment strategy is associated with a study claiming that it prolongs the survival time of patients. However, almost all patients are ultimately defeated by the tumor or the side effects, which has led to many questions about the effectiveness of cancer treatment. Thus, with all these advanced treatments, one may wonder why a significant reduction in cancer deaths has not occurred. Perhaps God knows the answer.

In short, huge challenges remain in exploring the most effective method for treating advanced melanoma, and this journey remains full of possibilities. In addition to the difficulties mentioned above, this study not only explores and verifies the differences in the expression of signature genes in patients with metastatic melanoma who received either chemotherapy or immunotherapy but also provides insights into differences in signature gene in terms of biological function, gene mutation frequency, protein domain, and CpG methylation level. These findings could provide important clues for the discovery of new tumor immunotherapy strategies.

## Supplementary Materials

Supplementary Figures

Supplementary Tables
