# Supplementary material for: Differences in and verification of genetic alterations in chemotherapy and immunotherapy for metastatic melanoma
Source: Aging (Albany NY). 2021 Oct 21;13(20):23672–88. doi: 10.18632/aging.203640 (PMC8580330; doi:10.18632/aging.203640)
Supplement: Supplementary Figures [file aging-13-203640-s001.pdf]

SUPPLEMENTARY FIGURES

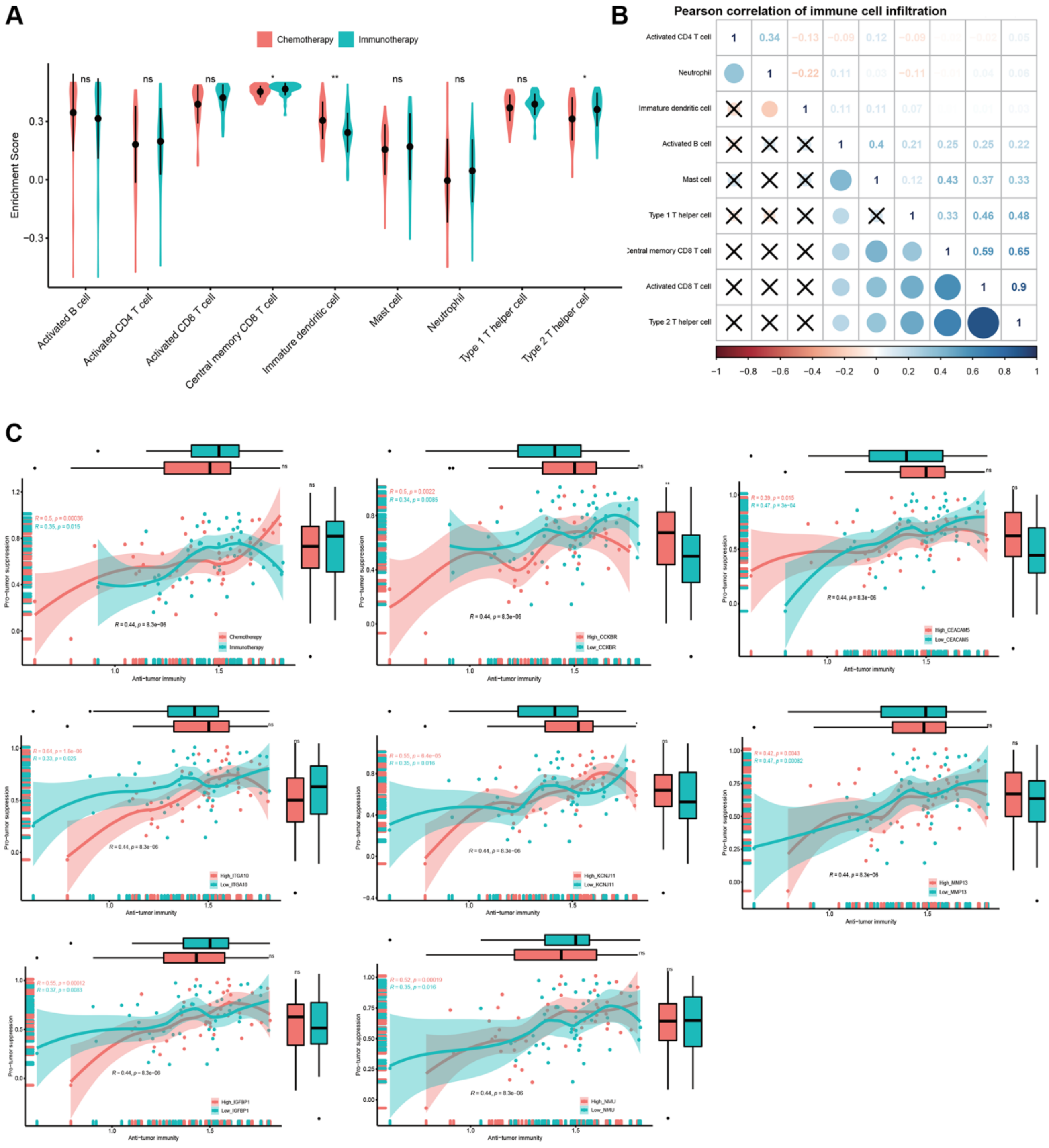

Supplementary Figure 1. Difference in DEGs-based immune cell infiltration. (**A**) Immune cell infiltration difference between chemotherapy and immunotherapy groups. (**B**) Pearson correlation of immune cell infiltration enrichment score. (**C**) Immune cell infiltration difference between low expression group and high expression group of signature genes.

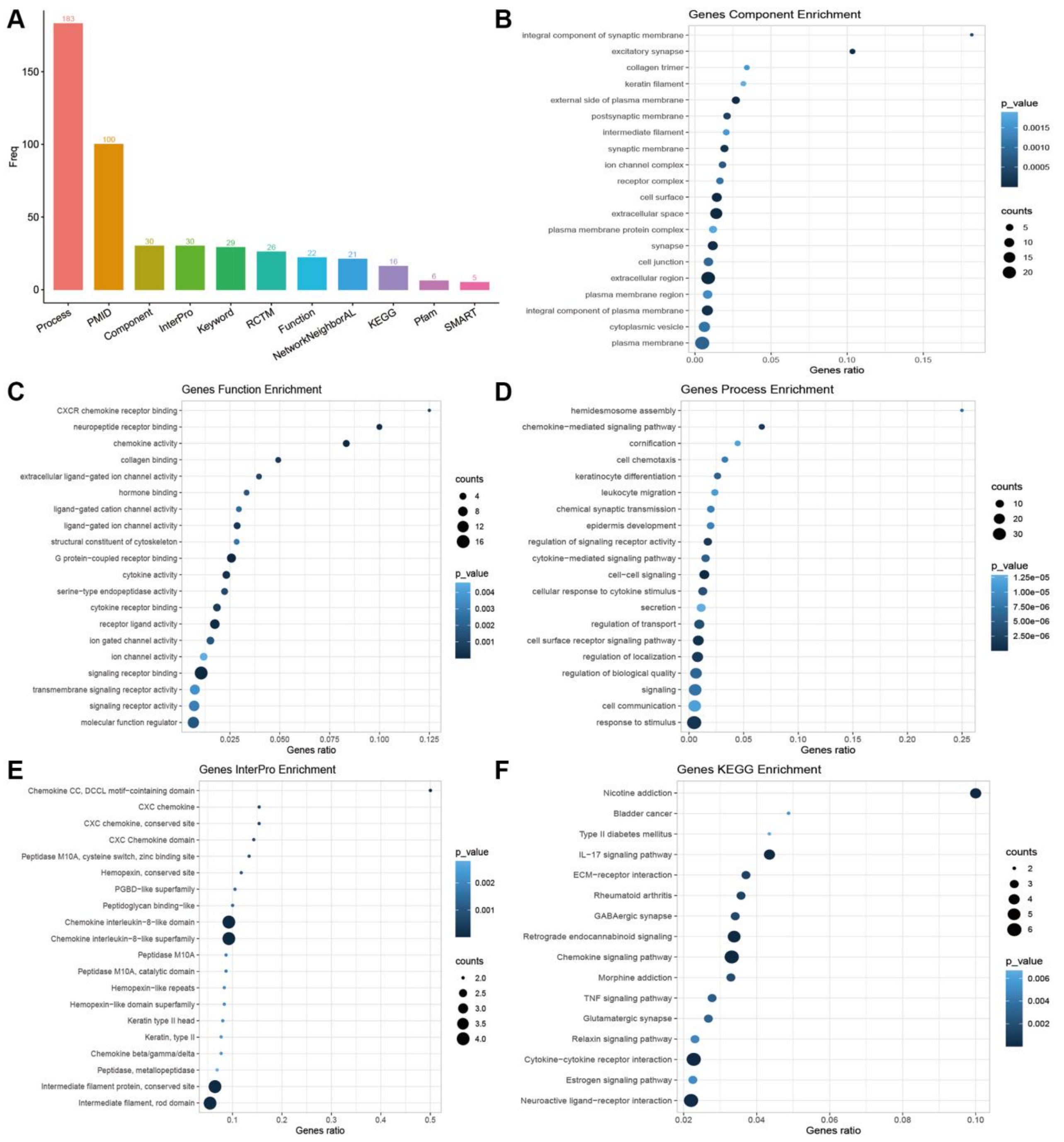

Supplementary Figure 2. Enrichment map of 50 important genes screened by PPI network. (**A**) Enrichment results of different functional distribution. (**B–F**) Dotplot of top 20 items in the enrichment results of cell composition, molecular function, biological process, integrated protein domains and KEGG pathway.

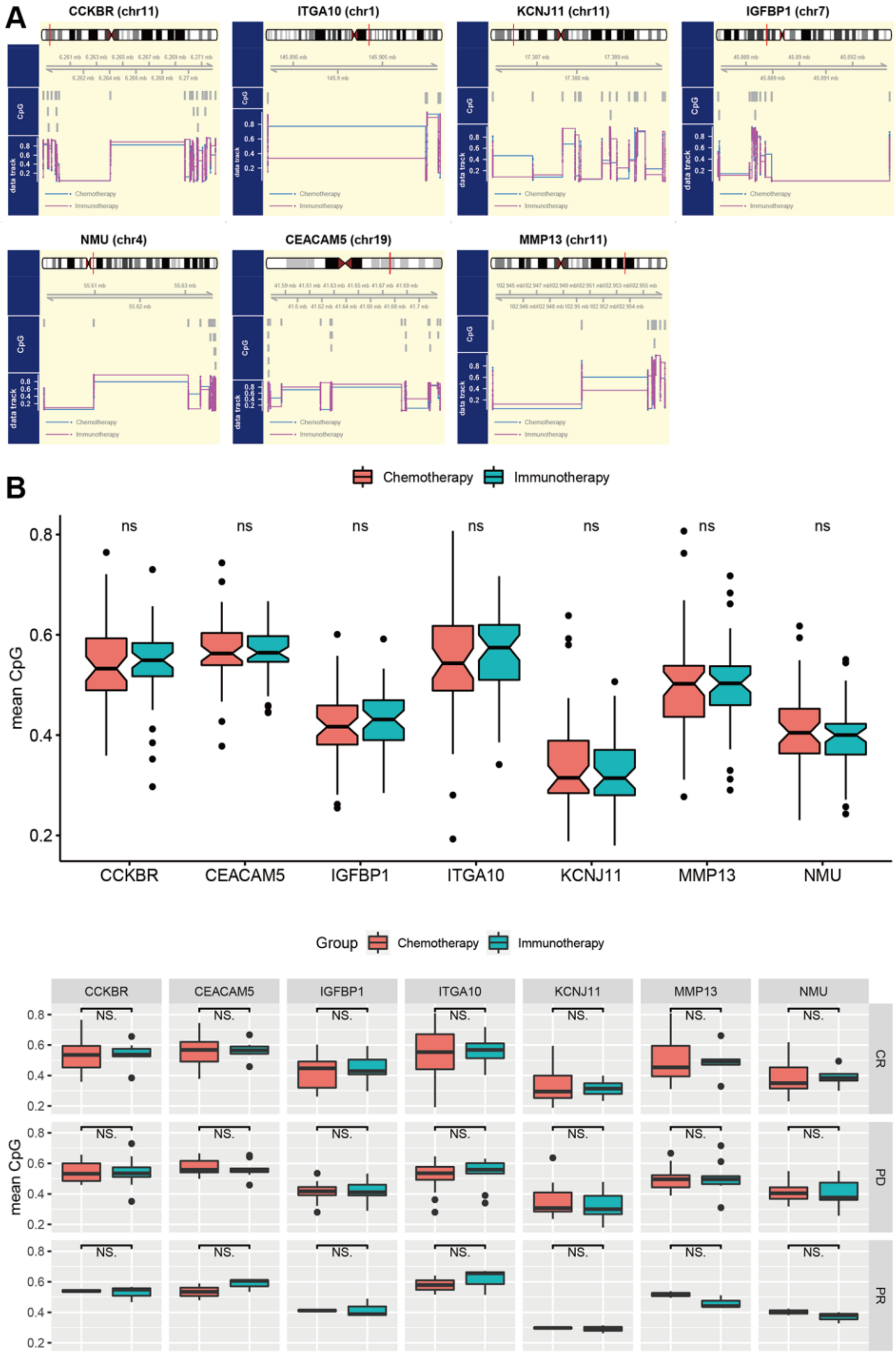

Supplementary Figure 3. Comparison of differences in CpG methylation of signature genes between the two groups. (**A**) Differences in the distribution of CpG methylation sites of signature genes between groups in chromosomes. (**B**) The difference in CpG methylation levels between the two groups, as well as in the complete response (CR), partial response (PR) and progressive disease (PD) subgroups.
