# Supplementary material for: Differences in and verification of genetic alterations in chemotherapy and immunotherapy for metastatic melanoma
Source: Aging (Albany NY). 2021 Oct 21;13(20):23672–88. doi: 10.18632/aging.203640 (PMC8580330; doi:10.18632/aging.203640)
Supplement: Supplementary Tables [file aging-13-203640-s002.pdf]

SUPPLEMENTARY TABLES

Supplementary Table 1. Primers of signature genes.

Symbols	Forward primers (5′ → 3′)	Reverse primers (5′ → 3′)	Amplicon size
CCKBR	GGGACACGAGAATTGGAGCTG	AACCGCCTTGCAGATGACG	249
ITGA10	AACATCACCCACGCCTATTCC	GTTGGTAGTCACCTAAGTGGC	207
KCNJ11	AGGTCCAAGTGACTATTGGCT	TCTGCACGATGAGGATCAGGA	81
IGFBP1	TTGGGACGCCATCAGTACCTA	TTGGCTAAACTCTCTACGACTCT	114
NMU	CTCAGGCATCCAACGCACT	GACTTGCCCAACTTCTGTGTC	136
CEACAM	CTGTCCAATGACAACAGGACC	ACGGTAATAGGTGTATGAGGGG	174
MMP13	ACTGAGAGGCTCCGAGAAATG	GAACCCCGCATCTTGGCTT	103

Supplementary Table 2. 50 important genes screened through PPI network.

Symbols	Gene names	log2 Fold Change	*P* value	PMID	Function
KLK3	kallikrein related peptidase 3	5.400	2.52E-23	10218588	angiogenesis
SLC17A6	solute carrier family 17 member 6	–4.658	6.89E-20	10820226	amino acid transmembrane transport
CHRNA1	cholinergic receptor nicotinic alpha 1 subunit	3.543	2.64E-12	10195214	action potential
DLK1	delta like non-canonical Notch ligand 1	–4.428	5.84E-12	10354070	molecular_function
SFTPA2	surfactant protein A2	3.575	6.19E-12	10781424	activation of innate immune response
FCER1G	Fc fragment of IgE receptor Ig	1.968	2.59E-10	10049942	cell activation
KRT14	keratin 14	–3.028	5.63E-10	10583131	molecular_function
SFN	stratifin	–2.655	3.62E-09	10524633	cell cycle checkpoint
ORM1	orosomucoid 1	2.314	5.94E-09	11027547	cell activation
COL17A1	collagen type XVII alpha 1 chain	–2.405	7.51E-09	10022517	immune system process
LMO1	LIM domain only 1	–2.211	1.28E-08	10603358	negative regulation of transcription by RNA polymerase II
COL6A5	collagen type VI alpha 5 chain	2.158	1.78E-08	14702039	molecular_function
CCKBR	cholecystokinin B receptor	1.682	7.36E-08	10100325	peptide receptor activity
ITGA10	integrin subunit alpha 10	2.459	1.05E-07	10702680	molecular_function
KCNJ11	potassium voltage-gated channel subfamily J member 11	1.682	1.19E-07	10093054	nucleotide binding
EREG	epiregulin	2.476	2.06E-07	10681561	reproduction
GABRB2	gamma-aminobutyric acid type A receptor beta2 subunit	1.984	2.93E-07	10023064	system process
ADCY8	adenylate cyclase 8	2.718	3.09E-07	10075700	nucleotide binding
KRT16	keratin 16	–2.625	4.24E-07	10521820	ameboidal-type cell migration
NRXN1	neurexin 1	–2.371	4.33E-07	11036064	cell morphogenesis
THBS1	thrombospondin 1	1.612	4.47E-07	101549	MAPK cascade
IGFBP1	insulin like growth factor binding protein 1	1.507	7.07E-07	10329650	regulation of cell growth
LIF	LIF interleukin 6 family cytokine	2.101	8.01E-07	10205054	reproduction
XAGE2	X antigen family member 2	2.286	2.59E-06	10197611	molecular_function
NLGN3	neuroligin 3	–1.557	3.51E-06	10767552	cell morphogenesis
CACNA1B	calcium voltage-gated channel subunit alpha1 B	–2.042	4.21E-06	10455105	nucleotide binding
KLRD1	killer cell lectin like receptor D1	1.584	4.30E-06	10023772	natural killer cell mediated immunity
CXCR6	C-X-C motif chemokine receptor 6	1.541	5.16E-06	10590105	G protein-coupled chemoattractant receptor activity
SYT3	synaptotagmin 3	–1.794	7.08E-06	10531343	cell morphogenesis
MCHR1	melanin concentrating hormone receptor 1	2.294	1.78E-05	10421367	peptide receptor activity
CTSG	cathepsin G	1.907	1.96E-05	10512690	lytic vacuole
IVL	involucrin	–2.015	2.22E-05	10908733	cornified envelope
KRT5	keratin 5	–2.084	2.25E-05	10234505	molecular_function
NMU	neuromedin U	–1.514	2.61E-05	10783389	temperature homeostasis
KRT6C	keratin 6C	–2.036	3.51E-05	11683385	molecular_function
SAGE1	sarcoma antigen 1	1.560	3.65E-05	10919659	cellular_component
LRRTM1	leucine rich repeat transmembrane neuronal 1	–1.967	5.79E-05	12477932	regulation of receptor internalization
TCN1	transcobalamin 1	1.801	8.91E-05	11373332	transition metal ion transport
TEX15	testis expressed 15, meiosis and synapsis associated	–1.854	0.000	11279525	reproduction
CXCL9	C-X-C motif chemokine ligand 9	1.541	0.000	10201891	syncytium formation by plasma membrane fusion
CCL20	C-C motif chemokine ligand 20	1.584	0.000	10064080	G protein-coupled receptor binding
MMP1	matrix metallopeptidase 1	–1.752	0.001	10224132	immune system process
DDX43	DEAD-box helicase 43	–1.587	0.001	10919659	nucleotide binding
CEACAM5	carcinoembryonic antigen related cell adhesion molecule 5	–1.590	0.002	10436421	immune system process
CXCL5	C-X-C motif chemokine ligand 5	1.666	0.002	10068592	molecular_function
GRIA2	glutamate ionotropic receptor AMPA type subunit 2	1.586	0.003	10027300	amyloid-beta binding
VGF	VGF nerve growth factor inducible	1.554	0.004	10381005	reproduction
CCL21	C-C motif chemokine ligand 21	–1.513	0.008	10201891	MAPK cascade
MMP13	matrix metallopeptidase 13	1.533	0.008	10074939	skeletal system development
EDN3	endothelin 3	1.997	0.009	10231870	MAPK cascade
